# The Adaptation of the Carbohydrate Counting Method Affects HbA1c and Improves Anthropometric Indicators in Patients With Diabetes Mellitus 2

**DOI:** 10.3389/fnut.2020.577797

**Published:** 2021-01-26

**Authors:** Adriana Beatriz Di Iorio, Domingo Orozco Beltrán, José Antonio Quesada Rico, María Concepción Carratalá Munuera

**Affiliations:** ^1^Food Agroindustry, Zamorano University, Yaguare Valley, Tegucigalpa, Honduras; ^2^Clinical Medicine, Miguel Hernández University, Alicante-Valencia, Spain

**Keywords:** diabetes mellitus 2, medical nutritional treatment, blood indicators of health, body indicators, hypoglycemia

## Introduction

The worldwide growth of diabetes mellitus (DM) has, in recent years, generated an exponential increase in associated comorbidities such as high blood pressure, cholesterol, and cardiovascular risk (CVR), with an increase in mortality in the population (1). At the same time, the global DM pandemic increased by 75% in recent decades, with a large proportion of affected individuals spanning all age groups from 1988 to 2010 (2). In the Americas, its prevalence has increased from 5 to 8.3% in recent years, particularly in Honduras, where 6% of individuals over 20 years of age have DM (3). As such, DM increases health care costs in low- and middle-income nations (4).

Diagnosing diabetes mellitus type 2 (DM2) has changed since the inclusion of glycosylated hemoglobin (HbA1-c), as it is ≥6.5% in DM2. The criterion for fasting glucose is ≥126 mg/dL, whereas glucose at 2 h is ≥200 mg/dL (5). A hyperglycemic state can lead to an underlying prothrombotic environment, an overactivation of the coagulation cascade, fatal thromboembolic complications, and, eventually, increased mortality in DM patients (6). Medical treatment focuses on three pillars: drugs, nutrition, and education (7). The use of metformin acts as a standard pharmacological insulin used by patients to avoid weight gain (8). Nutritional medical therapy (NMT) prioritizes glycemic control and reduces comorbidities (7, 9). The diet promotion program is based on dietary guidelines, with group physical activity proving effective for predicting DM2 sowing but ineffective for long term benefits due to the lack of adherence (10). To this effect, the American Diabetes Association emphasized the need for individualized medical nutritional therapy (IMNT) (11).

Carbohydrate counting (CCHO) has been shown to be effective for glycemic control in diabetes mellitus type 1 patients when being intensively treated with insulin (12, 13). Carbohydrate counting considers the actual content of food consumed based on the individual's usual intake and coordinates insulin-glucose utilization so that both curves act as a single exponentially flattened growth curve (14). The resulting weight gain is a consequence of decreased urine sugar loss (15). Few studies have used carbohydrate counting in DM2 in the primary care setting, and although it showed improvements in HbA1c, compression of carbohydrate counting was considered difficult for participants (16). Given the paucity of evidence from randomized controlled clinical trials in Latin American for carbohydrate counting DM2 patients, this work aimed to evaluate the effectiveness of this medical nutritional treatment, which minimizes the risk of developing comorbidities and public spending on health care.

## Methods and Materials

### Study Type

The present work was a double-blind randomized controlled clinical trial. The allocation of the University School Hospital of Honduras was random. The study design was submitted and approved by the Biomedical Research Ethics Committee (IRB N°419-CGPGFCM/UNAH/2017) of the National Autonomous University of Honduras, on June 9, 2017. The doctors and nutritionists assigned to this study took an online ethics course titled “Human Subjects Research, IRB, Behavioral and Educational Focus” via the Collaborative Institutional Training program. As such, they were in compliance with the CONSORT checklist (17), which states the information to be included when reporting a randomized clinical trial. The study was carried out at the National Autonomous University of Honduras. The University School Hospital of Honduras has a specialized unit for the comprehensive care of DM patients and has recently created facilities conducive to the interdisciplinary medical-nutritional approach named “Model Center for Training and Comprehensive Care in Diabetes.” It has become the most prominent medical center in Honduras.

The working group was made up of the clinical epidemiology unit and the endocrinology unit belonging to the University School Hospital of Honduras. The director of the Model Center for Training and Comprehensive Care in Diabetes selected two doctors to adjust drug treatments and two nutritionists to apply and follow up with the carbohydrate (CCHO) count and current dietary recommendations (RDC). The working groups were trained separately for the application of nutritional medical therapies. The randomization of the participants was carried out by the head of the clinical epidemiology service. using the “random” function in Microsoft Excel.

### Sample Description

#### Inclusion Criteria

Participants with a DM2 diagnosis were selected based on clinical records, glycosylated hemoglobin ≥7% (not older than 6 months), aged between 18 and 65 years, insulin use between 1 and 10 years, no use of sulfonylureas, body mass index (BMI) <35 kg/m^2^, and waist–hip ratio ≥0.90 in men and ≥0.85 in women.

#### Exclusion Criteria

Patients excluded were those who had been clinically diagnosed as having cancer, chronic respiratory disease, pregnancy, cognitive impairment (e.g., dementia, amnesia, delirium), macroangiopathy (e.g., ischemic heart disease, stroke, peripheral vascular disease), microangiopathy (e.g., proliferative retinopathy or maculopathy, kidney failure grade IIb, III, or IV), amputations, temporary staff of the institution, and those with insulin use over 10 years.

### Sample Size

In 2016, the diabetic population of the University School Hospital of Honduras was 4,247 patients. In this study, 400 eligible patients in the endocrinology unit were selected. A mean value of 8.33% glycosylated hemoglobin was considered a regular value (11). For the sample size, we assumed risks foreseen in the current dietary recommendations of the control group (10%). The risk value of making an error was the conventional alpha of 5% (bilateral hypothesis) and beta of 20%. Participants (*n* = 62), per medical nutritional treatment, were adjusted for 15% loss and resulted in 71 participants per carbohydrate counting group for the current dietary recommendations.

**Figure 1:**
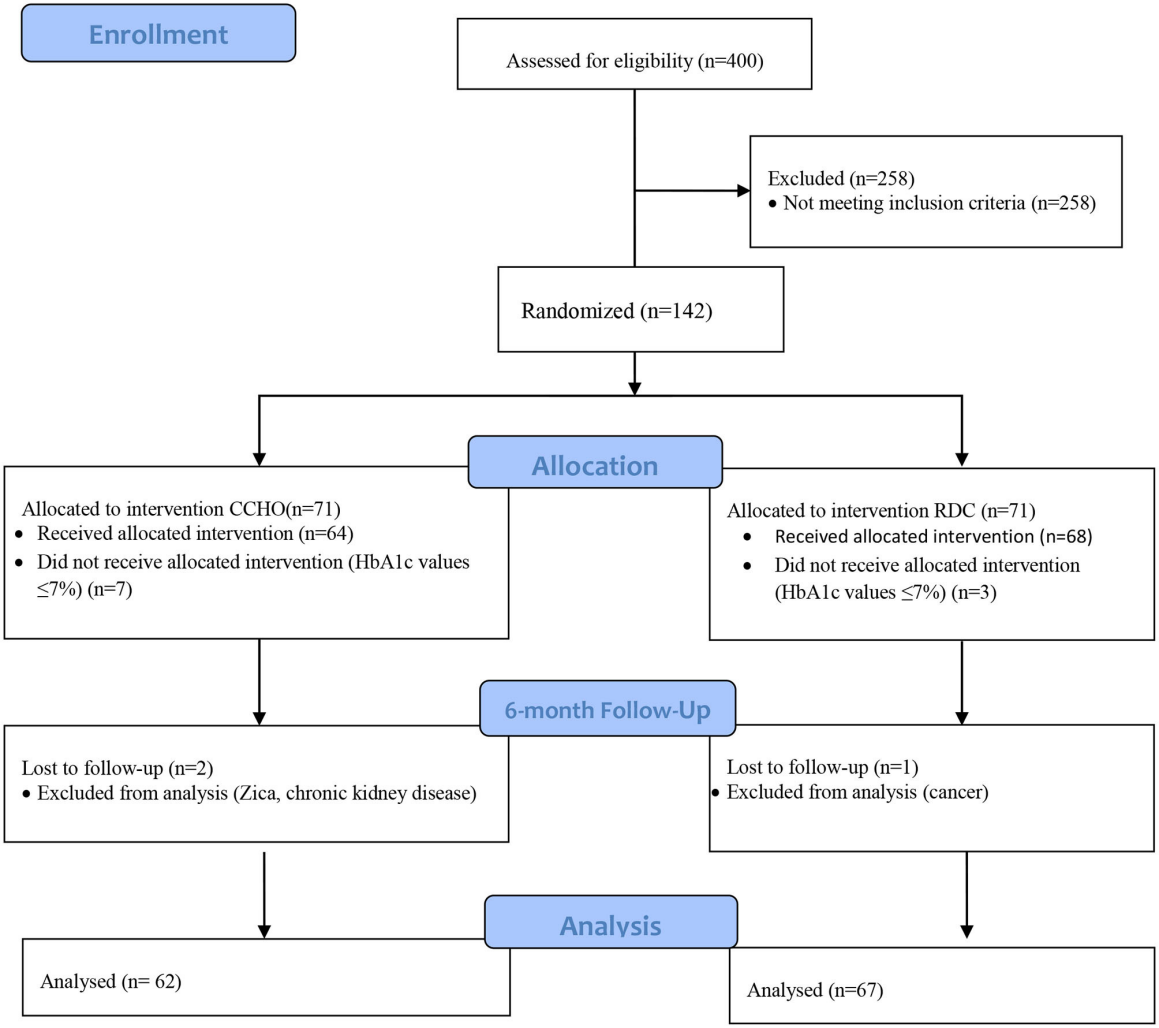
Flow chart of the selection process.

Figure 1 shows the flow chart of the selection process. The sample chosen was made up of participants registered with the endocrinology unit. In total, 400 participants were eligible, 258 participants did not meet the inclusion criteria, 142 participants were randomized, and only women decided to participate. During allocation, ten patients with glycosylated hemoglobin values ≤ 7% at the start did not receive allocated intervention. This left a total of 132 patients who met our inclusion criteria. During the 6-month follow-up, three participants were excluded because they had been diagnosed with cancer, chronic renal failure, and Zika, respectively. A total of 9.2% (*n* = 13) participants were excluded from the analysis. In total, 48.1% (*n* = 62) of participants remained on the carbohydrate counting diet, whereas 51.9% (*n* = 67) of participants followed their current dietary recommendations.

### Nutritional Medical Therapy Allocation

#### Nutritional Medical Therapy With Carbohydrate Counting

Participants adjusted to the insulin dose prescribed in the drug treatments (13, 14). The calorie requirements based on total energy expenditure were obtained (18). A reduction of 250 kcal/day to allow for adherence to treatment was applied. The nutritional medical therapy was delivered with a list of foods that contained the weight of carbohydrates and insulin (UI) dose, which was adapted to local culinary habits (forms of preparation and ways in which the food was cooked). Moreover, the nutritional medical therapy was adapted to the Central American food pattern (19).

#### Nutritional Medical Therapy With Current Dietary Recommendations

Participants were issued a brochure by the Honduran Ministry of Health to help better manage their diabetes mellitus type 2 (20). The brochure details glycemic index, fat content, and food preparation style but does not estimate caloric requirements nor provide food caloric adjustments.

#### Study Follow-Up

Participants in both nutritional medical therapy groups (i.e., carbohydrate counting and current dietary recommendations) took part in 30-min sessions in which they observed photographs that showed food portions, food fiber, types, and cooking techniques. Biochemical glucose, glycosylated hemoglobin, and anthropometric (i.e., weight, height, body mass index, waist circumference, hip circumference, and blood pressure) parameters were evaluated at 6 and 12 months of individualized nutritional therapy. Glucose, however, was evaluated monthly. At each visit, doubts were cleared up, and concepts were reinforced for each nutritional medical therapy.

### Data Collection

#### Biochemical Parameters

For both nutritional medical therapies the glucose (mg/dL) and glycosylated hemoglobin (%) blood parameters were collected the day of the appointment and analyzed at the University School Hospital of Honduras's clinical laboratory. Participants attended these sessions on an empty stomach and without having previously smoked for 8 h. The 3-mL blood samples obtained using venipuncture were stored in anti-clotting tubes labeled with the code of each participant. They were centrifuged with a Z-29 digital macro-centrifuge kit at a speed of 2,500–3,000 rpm for 5 to 10 min to separate the serum from the clot. They were incubated at 65–85°F ± 5°F (17–30°C ± 2.8°C) per h. Glucose and HbA1c values were analyzed using Siemens Dimension RxLMax equipment (Siemens Healthcare Germany 2010). HbA1c determination was performed using the ascendant/enzymatic endpoint method, with FlexR reagent cartridge reagents.

#### Anthropometric Measurements

On the same day of the biochemical measurements, we recorded the weight, height, waist–hip size, and blood pressure of participants in accordance with recommendations (21). They were weighed (kg) with precision class I equipment (seca 803). Height (recorded in cm) was measured using a stadiometer (seca 213). Body mass index was calculated in accordance with the standard formula (kg/m^2^) (22). Blood pressure (mmHg) was measured three times at an interval of 1 min between each measurement (23) using a digital automatic blood pressure monitor (OMRON M6 (HEM-7001-E (V); precision ± 3 mmHg). Therefore, we obtained the values with the means of the second and third measurements of systolic (SBP, mmHg) and diastolic blood pressure (DBP, mmHg).

#### Definition of Central Obesity

Waists and hips (cm) were measured with a flexible tape measurer (SECA 201). Pressure was avoided on the tissues. Central obesity, waist circumference (≥80 cm), and waist–hip ratio (≥0.85) were defined in accordance with World Health Organization (WHO) criteria (24).

#### Statistical Analysis

The results of each dietary treatment were expressed as mean ± standard error of the mean. The normality of the data was verified using the Kolmogorov-Smirnov test. The uniformity and variance were verified using the Bartlett test. An unpaired Student *t*-test was performed. Pearson correlations were analyzed to verify the association between HbA1c and anthropometric indicators. The data to compare experimental stages were processed using the Friedman test. Over time, we adjusted the mixed linear multivariate model for HbA1c, taking into account the possible effect of the explanatory variables. A stepwise variable selection process was carried out based on the AIC (Akaike Information Criterion), until the optimal model was reached. The optimal model is shown, with the coefficients, standard error, degrees of freedom, and the *t*-value and *p*-value associated with each coefficient of the fixed effects. The standard deviation and the correlation of the random effects (time) are also shown. We used SPSS software version 22 (IBM (International Business Machine), New York, USA) and the nlme package. The R statistical program was used for mixed models.

## Results

Table 1 shows the effect of carbohydrate counting and current dietary recommendations on anthropometric and biochemical values at 0, 6, and 12 months of treatment. Significant differences were observed between treatment for diastolic blood pressure, body mass index, waist, glycosylated hemoglobin, insulin units, and metformin doses. Diastolic blood pressure was reduced in CCHO from 82.73 to 77.05 mmHg (*p* = 0.001), while RDC did not obtain a reduction (*p* = 0.747). Changes in diastolic blood pressure values were observed between groups from 6 months of nutritional medical therapy with statistical significance at 12 months (77.03 mmHg CCHO vs. 80.87 mmHg RDC *p* = 0.019). Body mass index varied over time, with its highest efficacy at 12 months of CCHO (30.74 to 29.74 in CCHO vs. 31.21 to 32.06 in RDC *p* < 0.001).

**Table 1:** Effect of medical nutritional treatment vis-à-vis anthropometric and biochemical markers.

	**Nutritional medical treatments**			
	**CCHO**	**RDC**	**SEM ±**	***p*-value^**1**^**
**Diastolic pressure (mmHg)**				
Initial	82.73^a^	80.60	1.106	0.179
6 months	81.63^a^	79.79	1.151	0.264
12 months	77.03^b^	80.87	1.144	0.019
*p*-value^2^	(80.46) 0.001	(80.42) 0.747		
**Body mass index (Kg/m**^**2**^**)**				
Initial	30.74	31.21	0.377	0.384
6 months	30.41	31.63	0.390	0.028
12 months	29.74	32.06	0.409	<0.001
*p*-value^2^	(30.28) 0.173	(31.63) 0.311		
**Waist (cm)**				
Initial	96.24^a^	99.16	1.130	0.069
6 months	94.98^b^	100.16	1.143	0.002
12 months	92.16^c^	101.21	1.237	<0.001
*p*-value^2^	(94.46) 0.026	(100.17) 0.533		
**Hip (cm)**				
Initial	102.60	104.16	1.148	0.336
6 months	101.79	105.21	1.199	0.045
12 months	100.32	105.2	1.189	0.002
*p*-value^2^	(101.57) 0.309	(104.85) 0.694		
**Glucose (mg/dL)**				
Initial	175.16	173.53^b^	9.089	0.977
6 months	173.53	188.41^b^	7.594	0.190
12 months	166.44	205.76^a^	6.662	<0.001
*p*-value^2^	(171.71) 0.257	(189.23) 0.007		
**HbA1c (%)**				
Initial	9.54^a^	9.29^b^	0.175	0.262
6 months	8.97^ab^	9.93^a^	0.175	<0.001
12 months	8.20^b^	9.97^a^	0.161	<0.001
*p*-value^2^	(8.90) 0.050	(9.73) 0.004		
**Insulin (UI)**				
Initial	49.81	52.00	2.614	0.552
12 months	42.95	55.41	2.6154	0.001
*p*-value^2^	(46.38) 0.079	(53.7) 0.477		
**Metformin (mg)**				
Initial	1762.74	1882.11	87.659	0.343
12 months	1407.95	2103.33	70.359	<0.001
*p*-value^2^	(1585.34) 0.003	(1992.72) 0.048		

a,b *Means with different letters in the same column differ at p < 0.05*.

p-value^1^: Unpaired Student t-test.

*p-value^2:^ Friedman test*.

*CCHO: carbohydrate counting*.

*RDC: current dietary recommendations*.

*SEM: standard error of the mean*.

Participants' waists achieved reductions of 1.143 cm at 6 months for CCHO and RDC (*p* = 0.02); however, at 12 months, waists began showing a marked trend, with a reduction of 1.23 cm in CCHO (*p* < 0.01). As a biochemical indicator, glycosylated hemoglobin showed an intergroup reduction at 6 and 12 months (*p* = 0.05). The intergroup (*p* < 0.01) compared with the RDC showed an increase in its values at 6 and 12 months (0.69%). The insulin and metformin values for the CCHO behaved with a reduction of administered units for the pharmacologic insulin intergroup (6.85, *p* < 0.001). Moreover, there was a decrease of 354.79 mg metformin at 12 months of nutritional medical therapy with a *p* < 0.001 difference between groups.

Table 2 shows a linear regression between HbA1c, BMI, waist, hip, glucose, insulin, and metformin. The β_*i*_ coefficient associated with each variable shows the average change in HbA1c by a one-unit increase in the model variable. In this way, in the RDC group, hemoglobin decreased by 0.12 (β of time factor 2) and 0.45 units (β of time factor 3) when passing from the basal level at 6 and 12 months, respectively. In the CCHO group, the average decrease was 0.62 (0.12 + 0.50) and 0.95 (0.45 + 0.50) units at 6 and 12 months, respectively. The significant reduction occurred at 12 months (*p*-value < 0.001), not at 6 months (*p*-value 0.169). The model presented a good fit. There was no lack of normality or homoscedasticity.

**Table 2:** Linear mixed multivariate model of intercept and two random slopes.

**Fixed effects**	**β*_***i***_***	**Std. Error**	**DF**	***t*-value**	***p*-value**
(Intercept)	6.3366	0.4928	241	12.86	<0.001
Time factor (2)^*^	−0.1288	0.0933	241	−1.38	0.169
Time factor (3)^*^	−0.4568	0.1003	241	−4.55	<0.001
Group: CCHO	−0.5036	0.1688	124	−2.98	0.003
Educational level Complete high school or higher	0.5470	0.2293	124	2.39	0.019
SBP (mmHg)	0.0144	0.0056	241	2.56	0.011
Glucose (mg/dL)	0.0079	0.0008	241	10.00	<0.001
Calories (cal)	0.0002	0.0001	241	1.95	0.052
Carbohydrates (g)	0.0017	0.0007	241	2.31	0.022
Cholesterol (mg)	0.0008	0.0003	241	2.86	0.005

* *Time factor (2): 6 months of treatment; time factor (3); 12 months of treatment*.

*DF: degree of freedom*.

*CCHO: carbohydrate counting*.

*SBP: systolic blood pressure*.

## Discussion

DM2 patients who counted carbohydrates showed effective gains regarding the main anthropometric and biochemical indicators during 30-min monthly sessions for 12 months. Table 1 shows that this individualized nutritional therapy presents beneficial effects to improve the state of health to prevent cardiovascular risk, which exponentially impacts quality of life. This potential decrease in cardiovascular risk is measured by a significant reduction of diastolic blood pressure (5.5 mmHg) at 6 and 12 months of treatment. The DASH sodium trial showed positive effects in blood pressure after 4 weeks of nutritional medical therapy with sodium reduction from 12 to 6 g, and a decrease of 10 mmHg in 5 weeks (25). Intra-abdominal fat produces certain proteins and hormones such as adipocin, angiotensinogen, and cortisol, all of which cause inflammatory processes that lead to high blood pressure (26). However, for this reduction to be effective, it is necessary for declines to coexist in other indicators, such as body mass index and waist. As suggested by the American Diabetes Association, no studies have demonstrated the efficacy of DM2 patients counting carbohydrates in an individualized medical nutritional treatment.

The present work reduced body mass index in participants, who transitioned from obese to overweight. These reductions depended on the time between groups, i.e., 6 and 12 months (*p* = 0.028) and (*p* < 0.001), waist (4.08 cm) (*p* = 0.026), and hip (2.23 cm) at 12 months. As such, cardiovascular risk (26, 27) decreased. Our study corroborated what has been mentioned by other authors on the relationship between weight gain at the expense of visceral adipose tissue. Further, we noticed an increase in systolic blood pressure (25–27), where a reduction in these indicators had an impact on the reduction cardiovascular risk (28–30). Carbohydrate counting at 12 months had the potential to normalize and improve these indicators, as well as reduce cardiovascular risk by improving the life expectancy for individuals.

Another important point to highlight in the evolution of this disease and its impact on quality of life are the biochemical indicators of glucose and glycosylated hemoglobin. In this study, it is shown that carbohydrate counting at 12 months achieved comprehension and adherence (31, 32), as well as a reduction in both indicators. A prospective study showed that severe visceral, parenchymal, and generalized adiposity are accompanied by inflammatory, neurohormonal, vascular, and metabolic responses that converge in cardiac and renal damage. Hypertension and diabetes mellitus are pathologies that amplify and perpetuate cardiovascular risk (33). Fasting glucose decreased by 8.72 mg / dL. Yet without statistical differences over time, glycosylated hemoglobin reflected a marked reduction of 1.34% over time (*p* < 0.001). Associated with these indicators, decreases in 12-month insulin and metformin doses of 6.86 IU and 354.79 mg (*p* = 0.003). Further, we found that *p* = 0.001 for insulin and *p* < 0.001 for metformin between groups.

These results demonstrate that carbohydrate counting affected not only the aforementioned benefits but also health expenses at the individual level. From a public health perspective, metformin reduced body weight by 2.1 kg compared to individuals who only receive drug treatment with insulin (8). However, this result was inconsistent with the data of the present study, as the current dietary recommendations did not improve any of the previously mentioned indicators. Likewise, in accordance with the American Diabetes Association, which considers carbohydrate counting the standard goal when managing diabetes mellitus type 1, we emphasized individualized nutritional therapy for stability and improvement of glycemic control in the prevention of vascular complications with HbA1c values <8% (5, 11). The present work achieved a glycosylated hemoglobin value of 8.20%, where it was clearly demonstrated that carbohydrate counting was effective for DM2 patients at 12 months. Moreover, it improved all indicators associated with cardiovascular risk.

Insulin is necessary to metabolize carbohydrates, proteins, fats, and maintain a certain euglycemia after meals. The main goal of insulin treatment is to mimic the physiological pattern of insulin secretion for better glycemic control (34). To maintain basal metabolism and limit liver glucose production between meals, 0.5–1 unit/h of insulin is needed. One unit of insulin is released for every 10 g of carbohydrate in the postprandial phase of insulin secretion (the meal-stimulated phase), which causes the diffusion of ingested nutrients (mainly glucose) to the periphery (35). A healthy patient's insulin secretion normally takes place 5 min after food intake. However, for DM2 patients, the first phase of insulin secretion is completely absent. The second phase, which in healthy patients lasts 1 to 2 h until blood glucose is normalized, is reduced by 50% in DM2 (33). In accordance with the data found in this paper, there is an association between the variable HbA1c and the values of insulin. Therefore, this indicator can be said to reduce the pharmacological dose and have a beneficial effect on individual health, given that cardiovascular risk is the main cause of death.

Intervention programs for physical lifestyle changes, such as weight loss, are proven to be ineffective (10). Therefore, individualized medical nutritional treatment and drug treatments could be key to preventing and reducing mortality rates for DM2 patients (11, 36). Past studies have shown the negative effects of weight gain with respect to indicators of arterial pressure and cardiovascular risk, among other effects (37). Herein, we observed anthropometric indicators for current dietary recommendations in accordance with similar studies where the increase in pressure was positively impacted by weight, central fat, body mass index, and basal metabolic rate (38). Another study that examined women in middle age correlated diastolic pressure positively with weight, visceral adipose tissue, and other indicators (26, 39). These results coincided with the data found in current dietary recommendations, which clearly shows that these factors negatively impact cardiovascular risk.

## Conclusion

For DM patients, individualized medical nutritional treatment, i.e., carbohydrate counting, improved patients' reduction of their cardiovascular risk measured via anthropometric indicators: body mass index (*p* < 0.001) diastolic pressure (*p* = 0.019), waist (*p* < 0.001), biochemical/glycosylated hemoglobin (*p* < 0.001), and glucose (*p* < 0.001). A decrease in the pharmacological dose of insulin (*p* = 0.001) and metformin (*p* = 0.001) was demonstrated.

### Study Strengths and Limitations

Our findings should be considered in light of the benefits of metabolic enhancement and its cardiovascular implications. The nutritional education received at each 30-min meeting allowed individuals to understand the method. The portion graphs and equivalent food measurements, as well as the adaptation of the method to the Central American diet, allowed participants to attend monthly scheduled appointments. From a social and health perspective, implementing the model for DM2 patients will not only impact the quality of life (i.e., less expenditure on drugs such as insulin and metformin), but it will also create a lower rate of long-term complications. The non-digitization of medical records and low educational levels of participants prevented a deeper understanding of the study's purpose, delaying the start of sampling. There were no methodological limitations during the development of this study.

### Future Directions

Future studies should replicate the present work at a local multicenter level and regional level and assess the similarities of data with the present work. Future studies should also insist on the importance of the nutritionist's role in primary health care centers in order to make possible the use of the method in the most needed regions.

## Data Availability Statement

The original contributions presented in the study are included in the article/supplementary materials, further inquiries can be directed to the corresponding author/s.

## Ethics Statement

The study design was submitted and approved by the Biomedical Research Ethics Committee (CEIB), (IRB 419-CGPGFCM/UNAH/2017) of the National Autonomous University of Honduras (UNAH). The patients/participants provided their written informed consent to participate in this study.

## Author Contributions

All authors listed have made a substantial, direct and intellectual contribution to the work, and approved it for publication.

## Conflict of Interest

The authors declare that the research was conducted in the absence of any commercial or financial relationships that could be construed as a potential conflict of interest.
